# The role of basement membranes in cardiac biology and disease

**DOI:** 10.1042/BSR20204185

**Published:** 2021-08-26

**Authors:** Erin Boland, Fabio Quondamatteo, Tom Van Agtmael

**Affiliations:** 1 Institute of Cardiovascular and Medical Sciences, University of Glasgow, Glasgow, U.K.; 2 Department of Anatomy and Regenerative Medicine, RCSI, Dublin, Ireland

**Keywords:** basement membrane, cardiac function, cardiomyopathy, developmental biology, extracellular matrix, heart disease

## Abstract

Basement membranes (BMs) are highly specialised extracellular matrix (ECM) structures that within the heart underlie endothelial cells (ECs) and surround cardiomyocytes and vascular smooth muscle cells. They generate a dynamic and structurally supportive environment throughout cardiac development and maturation by providing physical anchorage to the underlying interstitium, structural support to the tissue, and by influencing cell behaviour and signalling. While this provides a strong link between BM dysfunction and cardiac disease, the role of the BM in cardiac biology remains under-researched and our understanding regarding the mechanistic interplay between BM defects and their morphological and functional consequences remain important knowledge-gaps. In this review, we bring together emerging understanding of BM defects within the heart including in common cardiovascular pathologies such as contractile dysfunction and highlight some key questions that are now ready to be addressed.

## Introduction

**Figure 1:**
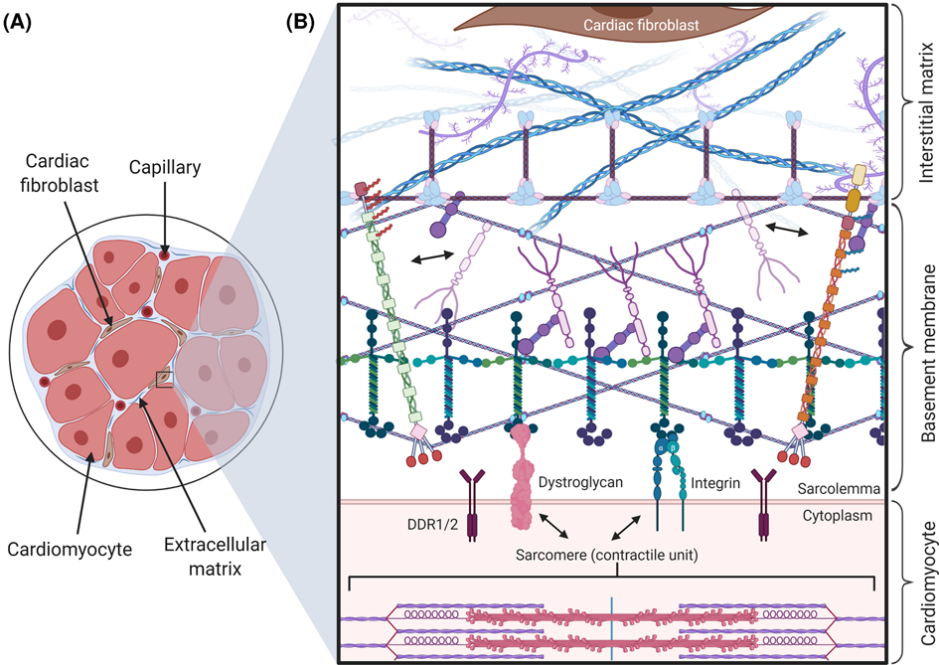
BMs within the heart (**A**) The ECM in the heart consists of BM and interstitial matrix that contains the fibrillar collagens (e.g. collagen I, III etc). BM (light blue) surrounds cardiomyocytes and microvasculature. Interspersed within the ECM are cardiac fibroblasts (brown) which are responsible for the secretion of many matrix components. (**B**) Magnified view of the BM as an interface between the interstitial matrix and cardiomyocyte sarcolemma. Various BM components interact with the cardiomyocyte sarcolemma via integrin, dystroglycan, and DDR receptors. Within the BM, laminin and collagen IV networks are assembled with perlecan and nidogen acting as ‘bridging molecules’ between these networks. Collagen XV and XVIII display their characteristic polarisation, linking the BM with the interstitial matrix. Collagen VI microfibrils interact with collagen IV, providing a physical link between the BM and fibrillar components of the interstitial matrix. For clarity, the lateral aggregation of collagen type IV is not shown and only limited examples of BM components and their interactions are represented. Abbreviation: DDR, discoidin domain receptor.

Basement membranes (BMs) are thin, sheet-like structures of the extracellular matrix (ECM) which serve as a barrier and anchorage point between the cell and interstitial ECM, and can influence cell differentiation, signalling, and migration in the vast majority of tissues [1]. Within the heart, BMs underlie the endothelial cells (ECs) and surround cardiomyocytes and vascular smooth muscle cells (Figure 1A). To date, relatively little is understood about the precise role of BMs in the heart. There is fragmentary knowledge throughout the literature from studies which have investigated BM components in isolation and how their absence affects cardiac development. Various animal models have been instrumental in understanding the distribution and temporal relationship between BM components and cardiac development. However, as these models often reflect multisystem disorders and/or are embryonic lethal, investigating adult cardiac function is often limited and mechanistic insight remains poor. For hereditary diseases affecting the BM, this knowledge is even more limited with the extent of cardiac involvement often being overlooked and the mechanisms which drive any pathologies almost completely unknown. In recent years, links between common cardiovascular pathologies and BM dysfunction have become more apparent and have suggested the importance of BMs in cardiac biology. However, significant gaps remain in our understanding, which if addressed, would shed light on how these pathologies develop and progress, and would reveal novel therapeutic targets. In this review, we summarise our current understanding of cardiac BMs and highlight questions which require further investigation and represent unexplored research avenues.

## General concepts of structure and functions of BMs

BMs have a wide array of biological functions, occur in almost all tissues, and underlie multiple cell types including epithelial and ECs, adipocytes, and myocytes. This physical demarcation compartmentalises tissues and cell types from one another (Figure 1A). BMs are crucial throughout embryonic development and tissue formation as they trigger and maintain cellular polarity [2]. They provide structural and mechanical support to tissues as BMs serve as an anchorage point between cells and the underlying interstitial ECM through various cellular interactions, and they also act as a reservoir for growth factors (Figure 1) [1,3]. For a more detailed overview on general BM function and biology, we refer the reader to a recent excellent review [4].

BMs generally comprise proteoglycans and glycoproteins with the primary components being type IV collagens, laminins, perlecan, and nidogens [5–10]. The composition and complexity of individual BMs is dependent on the tissue and cell type that they surround or underlie [11]. This compositional heterogeneity results in diverse cell–matrix interactions and multiple biological functions tailored to their location. Recent elegant single cell RNA sequencing analysis revealed that within the heart, different cell populations including cardiomyocytes, cardiac fibroblasts, vascular smooth muscle cells, and ECs contribute to the production of cardiac BM components [12]. Recent data from *Drosophila* development showed diffusive properties of some BM components and that components can be secreted and deposited by cells that do not reside on the BM [13]. This is also supported by elegant data from *Caenorhabditis elegans* where tagging of endogenous BM components revealed that some components, such as laminin and collagen type IV, form stable scaffolds within the BM while other components remain mobile, facilitating dynamic changes in BM composition over time [14]. It should, therefore, be noted that individual BM components expressed by a particular cell type could be incorporated into one or multiple BMs within the heart. The complexity and heterogeneity of cardiac BMs will require further in-depth assessment to understand how their composition relates to function. Revolutionary single cell RNA sequencing data of the adult heart have been compiled and are now readily accessible in various atlas formats [15,16]. In combination with proteomics and immunostaining data, these tools offer an incredibly useful resource that could be utilised to provide some overview regarding the composition of the different BMs within the heart and the putative cellular origin of several BM components within the heart.

## BM components in the heart

In this section, we will give a brief overview of the major BM components expressed within the heart and those in which an associated cardiac phenotype has been reported.

### BM collagens

Vertebrate genomes encode 28 types of collagen that vary in composition and biological activity [17], and have been divided into types based on their protein domain and supramolecular structures [18]. For this review, we will not cover all BM collagens but will focus on those for which a role in heart biology has been documented.

### Collagen IV

**Figure 2:**
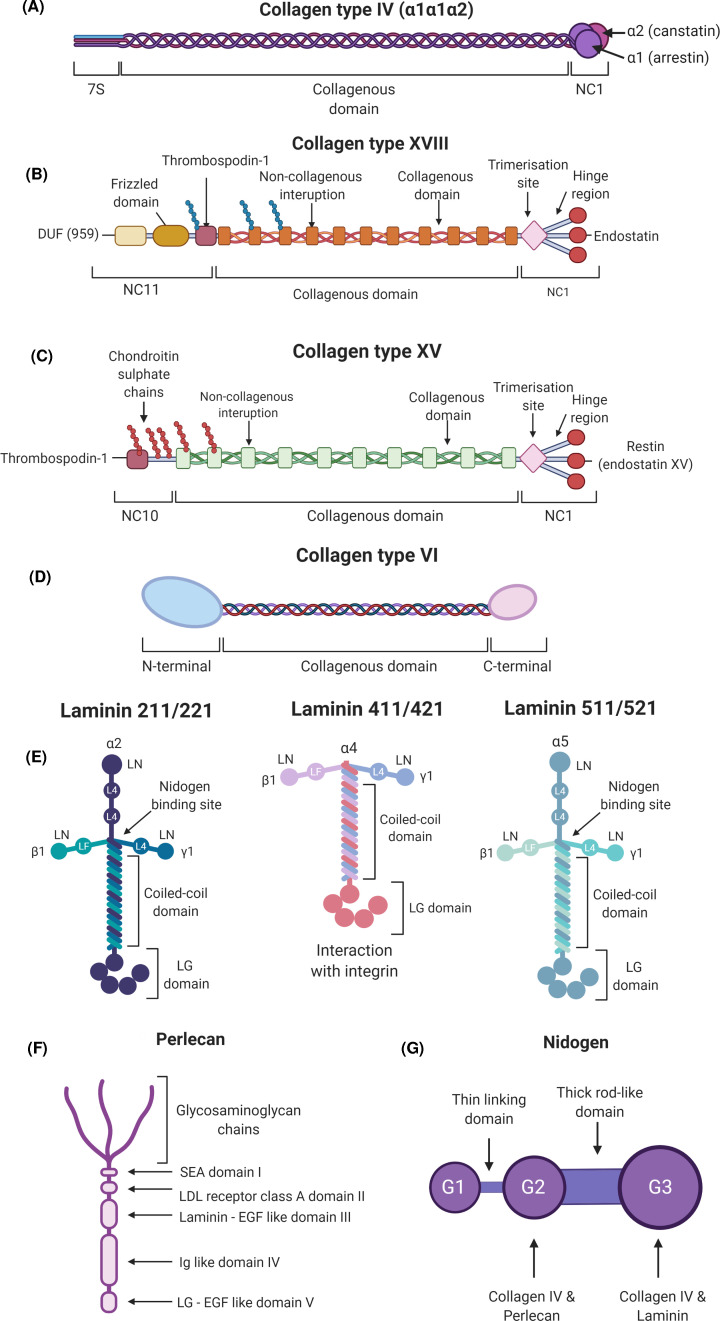
Major BM components of the heart (**A**) Protein domain structure of collagen type IV. Each α chain consists of a large central collagenous domain flanked by a N-terminal 7S domain and a C-terminal non-collagenous NC1 domain. (**B**) Collagen type XVIII (**C**) Collagen type XV. Collagen types XVIII and XV are structurally very similar, but vary in their glycosaminoglycan side chain composition with collagen XVIII binding heparan sulphate chains and collagen XV predominantly chondroitin sulphate chains. The collagenous domain of collagen type XVIII is flanked by a NC1 domain at the C-terminus which contains a trimerisation domain, a hinge region, and an endostatin peptide. (**D**) Collagen type VI α1(VI) and α2(VI) chains comprise a short triple helical collagenous domain flanked by globular N-terminal and C-terminal domains, with the C-terminal consisting of two von Willebrand factor type A domains. The α3(VI), α4(VI), α5(VI), and α6(VI) chains are structurally homologous. However, their N-terminus and C-terminus are larger and more variable than that of α1(VI) and α2(VI). (**E**) Laminin isoforms expressed within the heart. All laminin isoforms contain an α, β, and γ chain that interact to form the coiled coil domain with each chain providing the arm of the cruciform shape. Laminin proteins interact via their N-terminal domains (LN) to enable laminin network formation. The C-terminal of the laminin α chain contains five globular domains (LG) that mediate interactions with cell surface receptors such as integrin and dystroglycan receptors. Laminin isoforms that contain the shorter laminin α4 do not exhibit the traditional cruciform structure. Nidogen-binding sites are also indicated. (**F**) Protein domain structure of perlecan. Perlecan has five domains; Domain I at the N-terminal which contains a SEA (sperm protein, enterokinase, agrin) region and binds glycosaminoglycan chains. Domain II is structurally homologous to the low-density lipoprotein (LDL) receptor. Domain III contains laminin domain IV regions interspersed with epidermal growth factor repeats. Domain IV contains Ig like repeats. Similar to domain III, domain V contains laminin globular domains interspersed with epidermal growth factor repeats. BM components and cell membrane receptors that bind the different domains are also provided. (**G**) Protein domain structure of nidogen 1 and 2. The three globular domains of nidogen are linked by a thin and thick rod like domains. Other BM components such as collagen type IV, perlecan, and laminin bind the globular domains allowing for nidogen to act as a ‘bridging’ molecule between the BM networks.

Several collagens are localised specifically to BMs with the most abundant structural component of BMs being collagen type IV [19,20]. For more in-depth reviews on collagen IV and its role in human disease, we refer the reader to [21,22]. Briefly, collagen IV is a non-fibrillar, network-forming collagen, and vertebrates have six genes, *COL4A1–6*, each encoding its own α chain α1–α6(IV). In contrast with fibrillar collagens like collagen I, II, III and V, collagen IV contains interruptions to the Gly–Xaa–Yaa sequence in its collagenous domain (Figure 2A) [23]. These interruptions increase flexibility of the protomer and network, and can aid in specific heterotrimer interaction as well as increasing the aggregation propensity of the protein [24]. In the endoplasmic reticulum (ER), the α chains are extensively post-translationally modified hydroxylation, glycosylation and galactosylation [25]. Three α chains interact through their NC1 domains in specific combinations to produce three heterotrimeric protomers; α1.α1.α2(IV), α3.α4.α5(IV), and α5.α5.α6(IV). This is followed by triple helix formation in a zipper-like fashion in a C-terminal to N-terminal direction. Collagen IV protomers are then secreted from the cell and incorporated into the ECM [26]. In the BM, the 7S and NC1 domains are vital in forming the three-dimensional collagen IV network as the NC1 domains of two protomers interact to form dimers, and the 7S domains of four protomers to form tetramers (Figure 1B and Figure 2A). Combined this leads to the generation of a lattice style network [27]. The α1.α1.α2(IV) is the predominant isoform throughout development and in the majority of adult tissues, including the heart [28].

### Collagen type XV and XVIII

Collagen XVIII and XV are proteoglycans and belong to the family of multiplexin collagens that contain multiple triple helical collagenous domains interspersed with interrupting non-collagenous domains [18]. Similar to collagen IV, the interrupted nature of the collagenous domains of collagen XV and collagen XVIII provides increased flexibility compared with fibrillar collagens (Figure 2B-C) [29,30]. Collagen XVIII contributes to the structural integrity of the BM due to its polarised orientation within the BM [30] whereby its N-terminal end extends into the sublamina densa, where it interacts with the fibrillar structures of the interstitial matrix (Figure 1B) [31]. The endostatin peptide contained within the NC1 domain is involved in the interaction of collagen XVIII with other BM components such as laminins and perlecan, and has received much attention for its reported effects on angiogenesis (Figure 2B-C) [31,32]. These three BM collagens contain cryptic biologically active polypeptide fragments, called matrikines, that are released following proteolytic cleavage in the ECM. Some of these have anti-angiogenic properties which is not shared by the parental, uncleaved molecule. For collagen IV angiogenic inhibitors derived from the NC1 domain include arresten (α1(IV)), canstatin (α2(IV)) and tumstatin (α3(IV)) [33]. Endostatin is liberated following proteolytic cleavage of the hinge region of Collagen XVIII (Figure 2B) [34], while the structurally similar C-terminal region of collagen XV (Figure 2C) contains the restin or endostatin-XV peptide [35].

### Collagen type VI

Collagen type VI is encoded by six different genes (*COL6A1–A6*) each encoding a respective α chain (Figure 2D) [36,37]. Collagen VI is unique in its structure and assembly as the α chains interact in a α1(VI), α2(VI), α3/α4/α5/α6(VI) pattern to form a heterotrimeric protomer [38,39]. These protomers form dimers, and finally tetramers within the ER before being secreted into the BM where they form microfilaments [40]. This complex structure and assembly allows Collagen VI to interact with a variety of cell membrane receptors, interstitial matrix collagens and BM components, specifically collagen IV (Figure 1B) [41].

### Laminins

Laminins are, besides collagen IV, the other major network forming component of BMs (Figure 1B). The molecular structure of laminin and interactions with other BM components have been extensively described and for more in-depth reviews, please see [42,43]. The initial characterisation of laminins by Timpl and colleagues was fundamental in the initiation of BM research and was pivotal in our understanding of BM structure and biology [42,44–48]. To date, 16 laminin isoforms have been identified in vertebrates whereby each laminin molecule comprises an α, β, and γ chain (Figure 2E). However, within the heart the major isoforms expressed are LM-211, 221, 411, 421, 511, and 521 (Figure 2E). There are five α, three β, and three γ chains and each is encoded by an individual gene [49]. Most laminins are folded into cross-shaped heterotrimers, although truncated T-shaped laminins also occur (Figure 2E). The laminin amino-terminal (LN) domains on the short arms of the α, β, and γ chains are essential for laminin network polymerisation and self-assembly within the BM as well as interaction with other BM components such as nidogen (Figure 1B) [42,50,51]. The C-terminus of the α chain contains five globular LG domains that are responsible for mediating cell–matrix interactions (Figure 1B and Figure 2E) [52].

### Perlecan

Perlecan is an multidomain heparan sulphate proteoglycan which is expressed throughout embryonic development, particularly during the vascularisation of embryonic tissues and the developing skeletal system [53–56]. This large proteoglycan has five distinct domains that interact with other BM components and cell receptors to mediate cell adhesion and angiogenic activity (Figure 2F) [57]. Experiments in *Drosophila* demonstrated that perlecan deposition in BMs is dependent on collagen IV [13,58,59]. Once incorporated, perlecan can bind other BM components, i.e. collagen IV, nidogen, and laminin, to provide BM assembly and stability (Figure 1B) [46,60,61]. Perlecan also binds integrin receptors as it contains laminin-like domains interspersed with epidermal growth factor repeats that enable EC adhesion [62,63]. Finally, in addition to integrin binding, it interacts with dystroglycan receptors via its laminin-like globular domains [64]. The core protein and heparan sulphate side chains of perlecan sequester growth factors such as fibroblast growth factor-2 (FGF2), vascular endothelial growth factor (VEGF) and platelet-derived growth factor (PDGF) [65,66], ensuring a key role in mediating growth factor signalling.

### Nidogens

Nidogens, also referred to as entactins, are sulphated glycoproteins (Figure 2G) and vertebrates encode two nidogens, nidogen 1 and nidogen 2 that contain binding sites for laminin, collagen IV, and other proteoglycans including perlecan [67,68]. Similar to perlecan, based on binding other BM components, including collagen IV and laminins *in vitro*, nidogen is proposed to serve as ‘bridging molecules’ between collagen and laminin networks in BMs (Figure 1B). Recent work in *Drosophila* highlighted that the contribution of nidogen to coupling the collagen IV and laminin network, and BM assembly and stability varies between tissues [69]. Therefore, the exact nature of these interactions and the role of nidogen in BM assembly and stability *in vivo* remains to be fully determined.

## Impact of BM disruption on cardiac development

To understand the role of BM in cardiac development, many studies have investigated effects of mutations in various BM components and how morphological changes to the BM during cardiac development translates into structural abnormalities in the embryonic heart. Based on these studies, the BM has been implicated in various key stages of cardiac development including cardiomyocyte differentiation and homeostasis, cardiac morphogenesis, endocardial cushion and atrioventricular valve development, stability of the microvasculature and angiogenesis. We refer the reader to several excellent recent reviews, which describe cardiac development events and the roles of ECM components during these processes in extensive detail [70–73]. Here, we will discuss the roles of BM components on cardiac morphogenesis that have been investigated in various transgenic models.

## Cardiomyocyte differentiation and homeostasis

Cardiogenesis relies on intracellular signalling to induce cardiomyocyte differentiation and activation of cardiac-specific gene expression within cardiac progenitor cells (CPCs). BM components such as laminins and collagen type IV localise to the CPC ECM niche in the developing mouse and human heart [74]. Specifically, *in vitro* three-dimensional models enriched for collagen type IV show increased incorporation of murine CPCs but the functional relationship and mechanisms which contribute to this expansion remain unclear [74]. Several laminin isoforms are differentially expressed regionally and throughout developmental stages. Laminin α1β1γ1 (LM-111) is the most widely expressed isoform throughout embryonic development. The role of the γ1 subunit on the differentiation and functionality of cardiomyocytes has been investigated *in vitro* using *LAMC1* knock-out murine embryonic stem cell-derived cardiomyocytes. Surprisingly, sarcomeric arrangement was not affected in these cardiomyocytes despite a lack of a BM, which suggests that although laminin deposition correlated with sarcomere development, functionally it may not have a direct role [75–77]. Recent *in vitro* work suggested LM-511/521 as a driver of cardiomyocyte differentiation from pluripotent stem cell-derived cardiomyocytes [78]. However, it remains to be determined if this mechanism also influences *in vivo* cardiomyocyte maturation.

### Determining cardiac morphology

The morphological events involved in cardiac development are dependent on cell migration, differentiation, cavitation, apoptosis, cell–cell interactions, and cell fusion [79]. Many of these rely on ECM remodelling and so it is vital to understand how the BM is specifically involved in the regulation of these events, as their disruption often leads to congenital heart defects.

Perlecan expression is particularly prominent during the early stages of cardiovascular development [53], which is reflected with Perlecan (*Hspg2^−/−^*)-deficient mouse embryos displaying severe cardiac abnormalities at ∼10.5 dpc associated with embryonic lethality at this timepoint. Clefts formed in the myocardium and blood can no longer be contained within the ventricles and leaks into the pericardium, resulting in cardiac arrest (Table 1) [54,80]. Ultrastructural analysis revealed defective BMs with decreased incorporation of other BM structural components, leading to cardiomyocytes where the BM was disrupted and rudimentary, or completely absent. These findings indicate that perlecan is necessary to maintain the structural integrity of the BM and myocardium under mechanical stress. Mouse models have also been employed to investigate the role of nidogen. While the hearts and BM of nidogen 1 (*Nid1^−/−^*)-deficient mice generally appear normal due to a compensatory effect of nidogen 2 [81,82], complete nidogen deficiency in *Nid1^−/−^ Nid2^−/−^* mice leads to death shortly after birth with lung and heart defects whereby some of the hearts were dilated [83]. Reduction in trabeculation and compaction of the ventricular wall was also observed, which led to cleft formation and intramyocardial haemorrhage, similar to that of perlecan-deficient mice albeit at a later stage (Table 1) [84]. However, the severity of this phenotype was variable and ranged from a slight reduction in compaction to complete separation of myocardial muscle fibres [84].

**Table 1:** Summary of various murine knockout models of BM components and the observed cardiac phenotypes

Model	Cardiac phenotypes observed	References
***Lama4^−/−^* mouse**	- Rupture and dilation of the coronary microvasculature - Cardiac haemorrhage - Focal regions of fibrosis - Cardiomyocyte death	[113,115]
***Col4a1/Col4a2* double null mouse**	- Cardiac haemorrhage - Bleeding within pericardium - Dilation of blood vessels	[28]
***Col4a2^em1(IMPC^*^)^*^Wts^* mouse (homozygous knockout)**	- Double outlet right ventricle - Ventricular septal defects - Malformations of the great intrathoracic arteries - Abnormal regions of tissue development	[94]
***Col18a1^−/−^* mouse**	- Increased coronary vascular permeability and neovascularisation	[91]
***Col15a1^−/−^* mouse**	- Increased myocardial stiffness - Cardiomyocyte disorganisation - Myocardial capillary rupture - Increased coronary vascular permeability and narrowing - Decreased juvenile LV function	[125,174]
***Col6a1^−/−^* mouse**	- Improved cardiac function and reduced remodelling following MI	[182]
***Perlecan^−/−^* mouse**	- Myocardial clefting - Cardiac haemorrhage - Bleeding within pericardium - Pericardial thickening - Transposition of the great arteries - Hyperplastic conotruncal endocardial cushions	[54,107,127]
***Nid1/Nid2 double null***	- Myocardial clefting - Cardiac haemorrhage - Trabecular hypoplasia - Mild reduction in myocardial compaction	[84]

### Endocardial cushion and atrioventricular valve development

Endocardial cushions serve as the sites of atrioventricular valve and septa formation, and deviation of the aortic and pulmonary outflow tracts. This septation continues during development resulting in the four chambered heart structure while ensuring directional blood flow that by-passes the pulmonary circulation *in utero* via foetal shunts such as the foramen ovale, ductus arteriosus, and ductus venosus [85]. Within the developing mouse heart, collagen XVIII is primarily localised to the vasculature and endocardial cushions [86,87]. This expression is closely associated with the migrating mesenchymal cells during valve leaflet formation with *Col18a1^−/−^* mice displaying ultrastructural alterations of the BM surrounding the valve leaflets (Table 1) [88]. Nonsense mutations in the gene *COL18A1*, which encodes the collagen XVIII α chain, causes the clinically and genetically variable disorder Knobloch syndrome [89]. Knobloch syndrome patients develop severe ocular and cranial malformations such as myopia, retinal detachment and occipital encephalocele, but hypertriglyceridaemia and renal fibrosis have also been reported [31,90,91]. However, Knobloch is characterised by variable expressivity whereby the clinical features vary between patients [92]. For example, there has been a case report of a patient diagnosed with Knobloch syndrome presenting with a ventricular septal defect in addition to other features of the disorder [93]. Thus, while heart defects are not a major feature of Knobloch syndrome, these mutations may interfere with cardiac development in some cases. This variable disease expressivity could be due to genetic and environmental modifiers but to date there has been no analysis reported on these.

A role for collagen IV in cardiac development has also recently been described through the generation of a new *Col4a2*-deficient mouse model. Near absence of *Col4a2* in *Col4a2^em1(IMPC)Wts^* mice causes embryonic lethality post-organogenesis with extensive cardiovascular defects including double outlet right ventricle, atrial and ventricular septal defects, and various vascular malformations (Table 1) [94]. The delayed onset of embryonic lethality and milder defects compared with *Col4a1^−/−^ Col4a2*^−/−^ mice may be due to some residual *Col4a2* mRNA production [94]. Importantly, a role for collagen IV in heart development is also supported by case studies of patients with deletions of regions of chromosome 13 that include *COL4A1* and *COL4A2* that developed congenital heart defects including double outlet right ventricle, pulmonary stenosis, and both atrial and ventricular septal defects. This suggests *COL4A1* and *COL4A2* are candidate genes for congenital heart defects stemming from impaired endocardial cushion development (Table 2) [95–97], as supported by the data from the *Col4a2^em1(IMPC)Wts^* mouse [94]. Hereditary angiopathy with nephropathy, aneurysms, and muscle cramps (HANAC) syndrome patients with *COL4A1* glycine substitution mutations can develop mitral/aortic valve prolapse, with some requiring artificial valve replacement later in life (Table 2) [98–100]. A recent case study of a patient with a *de novo* glycine to glutamic acid mutation in the *COL4A1* gene was diagnosed with mitral valve regurgitation and the presence of an abnormal muscle bundle which obstructed outflow from the left ventricle upon echocardiogram assessment (Table 2) [101]. The data from mouse models and patients support that the reduction in extracellular collagen IV causes congenital heart defects affecting endocardial cushion formation, but the mechanisms for the adult phenotypes remain unclear.

**Table 2:** Cardiac phenotypes in patients with mutations affecting BM components

Gene affected	Mutation(s)	Reported/typical patient cardiac phenotypes	Reference(s)
***COL4A1***	p.P352L	Aortic valve insufficiency	[121]
	p.G498D, p.G498V, p.G510R, p.G525L, p.G528D, p.G624X	Supraventricular arrhythmia	[119,99,188]
	p.G749S, p.G618E	Mitral valve prolapse/regurgitation	[101,117,100]
	p.E1615K	Atrioventricular septal defects	[96,97]
	p.G148E, p.G209S, p.G696C	Foetal arrhythmia/abnormal cardiotocography	[189]
	p.G618E	Obstructive hypertrophic cardiomyopathy	[101]
	13q33.3q34 deletion	Double outlet right ventricle	[95]
	p.G118D	Dilated right atrium and ventricle Cardiomegaly Tricuspid regurgitation/dysplasia	[96]
***COL18A1***	ND	Ventricular septal defect	[93]
***COL6A1***	ND (UCMD)	Cardiac arrhythmia	[191]
	ND (Bethlem myopathy)	Progressive left ventricular dysfunction in pregnancy	[181]
	Trisomy 21	Various atrioventricular septal defects	[104]
***LAMA2***	p.T821P, p.C1469R, p.R1549X	Dilated cardiomyopathy	[148,147]
	p.H2627G, p.R1844S,	Palpitations	[190]
	p.R1350X	Mitral regurgitation	[190]
	p.T821P, p.C1469R, p.R1549X,	Conduction defects	[147,148]
	p.H2627G, p.R1029fs	Myocardial wall hypokinesia	[190]
***LAMA4***	p.D879H	Hypoplastic left heart syndrome	[146]
	p.D1309N	Infantile dilated cardiomyopathy	[146]
	p.P943L, p.R1073X	Severe adult dilated cardiomyopathy	[145]
***LAMA5***	p.V3140M	Mild mitral valve insufficiency	[102]
***LAMB2***	p.R1539G	Double outlet right ventricle Hypoplastic left heart syndrome Septum secundum defects Mitral valve atresia	[103]
***LAMC1/NID1***	ND	AV canal defects Atrial and ventricular septal defects Pulmonary stenosis	[108]

The vast majority of these cardiac phenotypes have been established through imaging techniques such as echocardiography. In patient case reports/summaries of disease phenotypes, cardiac involvement is often not reported or not described in detail. **ND**, mutation information not described.

Mitral valve insufficiency and atresia have also been recorded in patients with *LAMA5* and *LAMB2* mutations [102,103]. Interestingly, a patient case study report of cardiac phenotypes associated with a *LAMB2* mutation also included hypoplastic left ventricle and double outlet right ventricle [103].

In early human cardiac development (week 5) collagen VI expression is localised to the endocardial cushion mesenchyme and is sparse throughout the developing myocardium [104]. As development continues, the expression at the endocardial cushions is more prominent and observed in the forming atrioventricular valve leaflets [104]. By week 11, expression is more intense in the subendocardial layer of the ventricles and atria [104]. Based on data from trisomy 21 patients, it has been proposed that overexpression of *COL6A1/2* is implicated in the development of congenital heart defects involving endocardial cushion-derived structures such as atrioventricular septal defects [104]. However, the underlying mechanisms remain unknown.

Perlecan-null mice also develop congenital heart defects including transposition of the great arteries (TGA) (Table 1) [105], whereby the connection of the arteries is switched and the thoracic aorta outlets from the right ventricle and the pulmonary artery from the left ventricle. TGA is often associated with other cardiac malformations such as ventricular septal defects, left ventricular outflow tract obstruction, pulmonary stenosis, and coarctation of the aorta. The majority of newborns with the TGA present clinically with cyanosis, however the severity is dependent on the extent of intercirculatory mixing and other cardiac pathologies [106]. In perlecan-deficient mice this developmental defect is associated with an increase in the cardiac mesenchyme, which forms the endocardial cushions, that leads to impaired rotation of the developing outflow tract and septation of the aortic and pulmonary artery [107].

Finally, mutations in *NID1* and *LAMC1* have been associated with a variety of severe congenital malformations, which are collectively referred to as Dandy–Walker syndrome [108]. This condition can result in variable cerebellar hypoplasia, meningeal anomalies, and occipital skull defects. However, an analysis of 148 cases identified various cardiac defects in 16.7% of these cases, all of which stem from impaired endocardial cushion development, i.e. atrial and ventricular septal defects and pulmonary stenosis [109]. However, little is known regarding the mechanism of *NID1* and *LAMC1* mutations and how they lead to the congenital heart defects in a subset of Dandy–Walker syndrome patients.

### Structural integrity of microvasculature and angiogenesis

Coronary vascularisation takes place following the compaction of the myocardium that produces trabeculae which are thick column-like ridges of myocardial tissue [110]. The deposition of BM components, as confirmed by immunolocalisation within the rat heart, precedes vessel tube formation, which suggests that the BM organisation provides a scaffold for the newly formed myocardial vessels [111]. Gene expression analysis revealed that the most abundant laminin network within the heart consists of LM-211 and LM-221 [112]. LM-511 and LM-521 isoforms are also expressed and are primarily within the larger coronary vessels [113,114]. Other laminins that are highly expressed during cardiac and muscle tissue development are LM-411 and LM-421 [47], suggesting a key role for laminin α4. *Lama4* knock-out mice progress through development, with the majority surviving birth. However, mutant mice display rupture and dilation primarily affecting the microvasculature which led to haemorrhaging throughout the embryo, including the heart (Table 1) [113]. This microvascular disease leads to hypertrophic cardiomyopathy associated with focal regions of fibrosis and cardiomyocyte death [115].

Monogenic COL4A1 syndrome due to mutations in *COL4A1* or *COL4A2* is a complex multisystemic disorder that includes ocular defects, intracerebral haemorrhage, cerebral small vessel disease, kidney disease, and muscle cramps [10,20–22,99,116–121]. While laminins have been implicated in the primary organisation of the cardiac BM in *Drosophila* and are critical for initial BM development, collagen IV is required to provide stability to newly arranged BMs [10,28,122,123]. Despite the detrimental effects on cardiovascular and cerebrovascular development in collagen IV-deficient mouse embryos, collagen IV is not required for establishing BMs in the developing embryo [28]. However, in mice, the absence of collagen IV causes the BMs to be rudimentary in nature without a consistent arrangement and they do not fully surround the cardiomyocyte perimeter (Table 1) [28]. Similar to the *lama4*^−/−^ mice, and perhaps not surprising given intracerebral haemorrhage due to *COL4A1* or *COL4A2* mutations, *Col4a1^−/−^ Col4a2*^−/−^ mice display bleeding from the heart and vascular defects which are thought to be due to vascular fragility driven by decreased BM stability and defective cell–cell contacts [28].

Collagen XV is highly expressed within the vasculature and musculature of cardiac and skeletal muscle [124–126]. In contrast with many knock-out mouse models of BM components, *Col15a1^−/−^* mice are viable [125], but develop a complex phenotype with skeletal and cardiac myopathies (Table 1). Cardiovascular defects occur in adult mice whereby the microvasculature displays luminal narrowing due to EC folding and swelling [125], with increased vascular permeability and capillary rupture (Table 1).

It is therefore clear that various BM components contribute to multiple aspects of cardiac development such as laminin and collagen which have influence on cardiomyocyte differentiation and microvascular structure. However, there are also defects which are unique to individual components, for example TGA in perlecan null mice relating to issues with outflow tract development [127]. It is yet to be understood what phenotypes result from general BM defects and which are due to impaired function of individual components. Future work could shed light on this by employing new cardiac-specific knockout models that negate any systemic effects associated with the wide expression pattern of BM components. These could also be used to disentangle roles in cardiac development versus adult heart function, and their mechanistic basis.

## Impact of BM disruption on the adult heart

Adult cardiovascular and cardiac diseases are the leading cause of death, place a significant burden on society, and their global prevalence has doubled since 1990 to 523 million in 2019 [128]. Increased understanding of pathomolecular disease mechanisms stands to underpin the development of novel treatment approaches. The ECM plays a critical role in cardiac homeostasis and injury, whereby almost all attention is focused on the interstitial matrix [70]. In this section, we review the roles of the BM within the adult heart as this remains under-explored. Specifically, we focus on the contribution of the BM to cardiomyocyte function and how this is affected in various disease states. We have broadly broken this down into three key aspects of adult cardiac function: mechanotransduction via BM interactions with the cardiomyocyte, systolic and diastolic function, and finally ion transport and cardiac rhythm.

### Mechanotransduction via BM interactions with the cardiomyocyte

**Figure 3:**
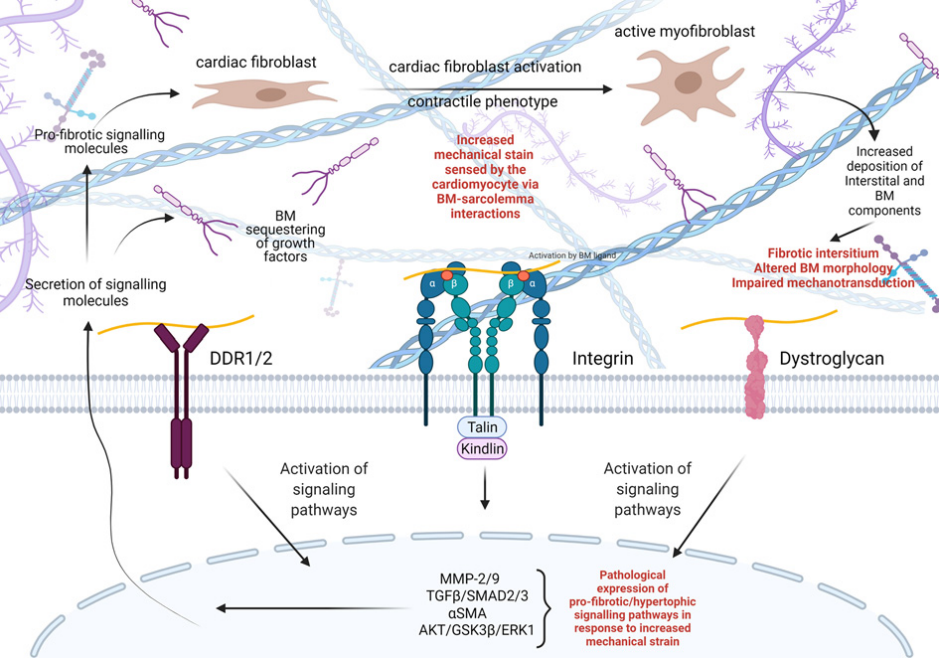
Influence of mechanical stress on BM composition and remodelling Mechanical strain on the heart is sensed by three major BM receptors present on the cardiomyocyte sarcolemma, DDR1/2, integrins, and dystroglycan receptors. These receptors are activated by interaction with various BM components, which results in intracellular signalling cascades. These can have a pro-fibrotic/hypertrophic influence on cardiac fibroblasts that undergo a phenotypic switch to a myofibroblast. In this state, the myofibroblasts increase matrix production including BM components producing a reciprocal feed–forward loop, which can become pathological leading to a fibrotic cardiac ECM and altered BM morphology. This impairment in cardiomyocyte–BM interaction further leads to activation of pathological signalling within the heart ultimately resulting in impaired mechanotransduction and cardiac function.

The composition and arrangement of the interstitial ECM is closely related to the mechanical properties of the heart. However, what is less well understood is how the composition and remodelling of the BM may influence the way cardiomyocytes are subjected to and respond to mechanical stress [127,129]. This stress activates signalling cascades in the cardiomyocyte, which in turn regulate the production of both interstitial ECM and BM components in a reciprocal nature (Figure 3) [130,131]. Ultrastructural analysis of failing hearts revealed that BM remodelling contributed to disease progression with disruption in myocyte tethering, ultimately leading to the inability to withstand increased afterload and mechanical stretch [132]. Reducing the mechanical strain on the heart through left ventricular assistance device saw a restoration in BM organisation and improved clinical outcomes for patients [133]. Together, these studies highlight the importance of the preservation of BM structure and myocardial stability. The BM provides this stability via interactions with several BM receptors present on the cardiomyocyte sarcolemma; integrins, dystroglycans, and discoidin domain receptors 1/2 (DDR1 and DDR2) (Figure 3).

Integrins are heterodimeric transmembrane glycoproteins comprising an α and β subunit (Figure 3) [134]. Upon binding with BM ligands such as collagen IV and laminin, integrin receptors cluster allowing for ‘outside-in’ signalling, which results in the interaction with cytoplasmic signalling and adapter proteins such as integrin linked kinase, focal adhesion kinase, paxillin, and talin [135,136]. Cardiomyocytes highly express integrins α_7_β_1_, α_5_β_1_, and α_1_β_1_, and to a lesser degree integrin α_3_, α_6_, α_7_, α_9_, and α_10_ [137]. Integrin receptors in striated muscle mainly localise to costameres, which are submembranous complexes that stabilise the link between the ECM and the cardiomyocyte sarcolemma at the sarcomeric z-line, and to the intercalated disks [138–140]. In the adult heart, integrins sense mechanical load on the ECM and convert it into an intracellular biochemical processes referred to as mechanotransduction [141] (Figure 3). The importance of integrin function in cardiac biology is further illustrated by the association of perturbed integrin expression with cardiovascular disease states, such as cardiac hypertrophy, heart failure, cardiac fibrosis, and contractile dysfunction [142–144]. Mutations affecting integrin interaction specifically with laminin-α4 (*LAMA4*) and laminin-α2 (*LAMA2*) have been identified as a genetic basis for the development of severe dilated cardiomyopathy, further emphasising the importance of cell–BM interactions in cardiac contractility [145–148].

Dystroglycans are the major non-integrin BM receptor on the cardiomyocyte cell membrane. They comprise an α and β dystroglycan domains, where α-dystroglycan is situated entirely within the ECM and serves as a laminin receptor [149,150] (Figure 3). β-dystroglycan is a transmembrane protein that interacts with α-dystroglycan and cytoskeletal proteins to form an adhesion complex with dystrophin and proteins such as dystrobrevin and syntrophin to stabilise the myocyte sarcolemma [151,152]. Dystroglycan-deficient mice are embryonic lethal with disruption to laminin and collagen IV deposition in Reichert’s membrane [153]. Further support for a role in BM assembly also comes from defective laminin and collagen IV deposition in dystroglycan-deficient embryoid bodies [154]. However, this BM disruption had no effect on cardiomyocyte differentiation within the embryoid bodies. Large myd mice are a model of congenital muscular dystrophy type 1D in which incomplete glycosylation of α-dystroglycan reduces its ability to bind laminin [155]. This is associated with focal myocardial damage, fibrosis, increased membrane permeability and elevated susceptibility to mechanical load stress [156]. As reduced glycosylation does not affect laminin deposition this supports that dystroglycan-laminin binding is critical for maintaining sarcolemma stability against mechanical strain [156].

DDR1/2 receptors are a class of non-integrin tyrosine kinase receptors which have extracellular ligands, specifically collagens (Figure 3) [157–159]. Both DDRs are activated by fibrillar collagens (types I, II, III, and V), however DDR1 also binds collagen IV, a property not shared by the DDR2 receptor which is mainly activated by collagen type X [159,160]. Upon binding of these collagens, the receptors become activated due to tyrosine autophosphorylation [161]. DDRs have been implicated in several diseases including cancer, atherosclerosis, and arthritis [159] and due to their influence over collagen fibrillogenesis and matrix remodelling, DDRs are key targets for several fibrotic disorders [162]. For example, DDR1 drives fibrosis in renal injury and can also be translocated to the nucleus to drive collagen IV expression [163]. This is also of interest in the heart as DDRs are markers of cardiac fibroblast activation [164]. In addition, DDR blockade increased murine-derived cardiac fibroblast differentiation and fibrotic lesion development post-myocardial infarction [165]. Interestingly, DDR2 knockout mouse revealed reduced heart mass, cardiomyocyte length, altered myocardial collagen deposition and cross-linking, as well as a reduced rate of collagen synthesis by cardiac fibroblasts [166]. DDR2 deficiency in mice also affected cardiac function with reduced cardiac contractility and rate of relaxation in response to ionotropic stimulation, mimicking increased cardiac stress [166]. Together, these data illustrate the importance of DDRs to normal cardiac function and matrix remodelling following cardiac injury. Combined, the complex interactions of BM components with each other and with cell receptors, provide a mechanism by which the BM modulates intracellular and extracellular processes that are at the basis of cardiac development and pathophysiology associated with focal myocardial damage, fibrosis, increased membrane permeability and elevated susceptibility to mechanical load stress [167] (Figures 1B and 3).

### Systolic and diastolic ventricular function

Given the contribution of the BM to the structural stability of the heart, we can begin to appreciate how impairments in the relationship between cardiomyocytes and the BM can be a feature in congenital and adult cardiac diseases. Heart failure is characterised by left ventricular insufficiency either due to a failure of the myocardium to contract (systolic dysfunction) or relax (diastolic dysfunction). Despite similar clinical presentations, systolic and diastolic dysfunction arise through different remodelling processes of the left ventricle [168]. BM components interact dynamically with transmembrane integrin receptors on cardiomyocytes, and the matrix–cell transduction of biomechanical stress causes hypertrophy via intracellular pathways such as melusin mediated AKT, GSK3β, and ERK1/2 signalling (Figure 3) [169]. Systolic dysfunction, or heart failure with reduced ejection fraction (EF), results from the reduced capacity of the left ventricle to contract and the ventricle wall becomes dilated. In this case, the collagen network becomes compromised by matrix metalloproteinase (MMP) degradation, leading to a decrease in the number of cardiomyocytes [170,171]. In systolic dysfunction, MMP expression is increased and results in the reduction in cross-links between collagen molecules [172]. Support also comes from a *Drosophila* model with advanced cardiac ageing in which BM thickness and expression of BM components such as laminin is increased with a reduction in lifespan and fractional shortening (FS), indicating systolic dysfunction and impaired contractility [173].

Functional analysis of *Col15^−/−^* mice identified the first impaired heart contractility due to deficiency of a BM component [125,174]. Interestingly, echocardiography revealed 1-month-old *Col15a1^−/−^* mice have a reduction in EF and FS indicative of systolic dysfunction [174]. With age these functional impairments disappear despite a persistence of morphological disorganisation of the cardiomyocytes. Interestingly, analysis of cardiac function in isolated hearts of older *Col15a1^−/−^* mice uncovered a marked reduction in left ventricular pressure in response to isoproterenol which pharmacologically increases heart rate. This indicates a decreased cardiac response following β-adrenergic stimulation, a feature which is associated with congestive heart failure and ageing [125,175,176]. These complex cardiac and microvasculature phenotypes in *Col15a1^−/−^* mice established that BM components are required for heart contractility and the maintenance of contractile function throughout life.

This is similarly reflected in patients with *COL6A1* mutations. Mutations in collagen VI result in two muscle-related disorders; Bethlem myopathy and Ullrich congenital muscular dystrophy (UCMD) [177]. Bethlem myopathy is characterised by progressive skeletal muscle weakness and joint contractures [178]. UCMD has similar features to Bethlem myopathy however, the disease is often more severe and earlier in onset [179]. It is currently thought that there is no significant cardiac component in Bethlem myopathy and UCMD [180]. However, intriguingly, progressive left ventricular dysfunction was noted in a patient diagnosed with Bethlem myopathy during pregnancy, raising potential implications for early delivery [181], and perhaps suggesting that collagen VI mutations may impact the ability of the heart to cope with the extra stress and workload in a progressive degenerative manner. The role of collagen type VI in myocardial infarction and cardiac function has also been explored in the *Col6a1^−/−^* mouse. Interestingly, following cardiac injury, adverse remodelling was reduced in knock-out animals suggesting collagen type VI contributes to the initial injury response [182].

Diastolic heart failure, or heart failure with preserved EF, differs from systolic dysfunction as the myocardium cannot relax efficiently leading to an increase in end diastolic pressure. Unlike systolic heart failure, diastolic heart failure often occurs when the ventricular wall is thickened as the cardiomyocytes undergo hypertrophy. However, diastolic dysfunction can also arise from other aetiologies including restrictive cardiomyopathy, constrictive pericarditis, and tension pneumothorax. In addition, the interstitial ECM undergoes remodelling as the production of fibrillar collagens (chiefly collagens I and III) increases, which is traditionally what is referred to when describing adverse ECM remodelling. Interestingly, and less frequently discussed, is that the expression of BM components such as collagen IV, laminin and fibronectin are also increased which contributes to the development of myocardial fibrosis and contractile dysfunction [183]. Models of prolonged supraventricular tachycardia (SVT) in pig hearts allowed for assessment of how cardiomyocytes, their BM and interstitial ECM respond to increased mechanical strain. SVT is an abnormally fast heart rhythm from which the source of improper electrical activity arises from the atria that results in reduced cardiomyocyte contractility and decreased ventricular filling time [184]. The anchorage of the cardiomyocytes to the BM is impaired as the sarcolemma fails to make complete contact with the interstitial ECM [185]. Following chronic SVT, the connection between interstitial collagen and the BM are weakened [186]. In addition, cardiomyocytes in systolic heart failure ‘slip’ due to reduced sarcolemma attachment to BM components which no longer provide structural support to the cardiomyocytes. The cardiomyocytes also become elongated, suggesting that the BM may play a role in maintaining correct cellular dimensions of the cardiomyocytes when under mechanical stress [185]. At the gross anatomical level this systolic dysfunction causes the left ventricular wall of these animals to dilate and to become much thinner and prone to rupture, meaning these hearts do not have the capacity to contract and cope with increased systolic pressures [187].

### Ion transport and cardiac rhythm

**Figure 4:**
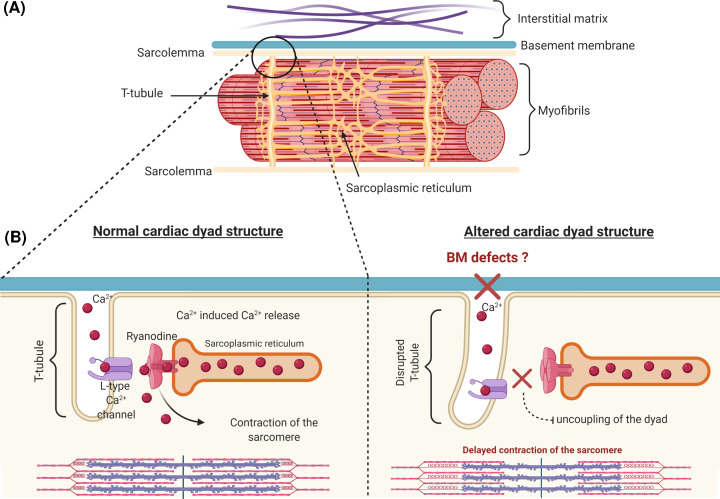
Structure of the cardiac dyad (**A**) Overview of the structure of the t-tubule (pale yellow), BM, ECM and myofibril. (**B**) T-tubule and interaction between the sarcolemma and the sarcoplasmic reticulum of the cardiomyocyte. In non-diseased states, the L-type voltage gated calcium channels present on the sarcolemma are maintained in close proximity to the ryanodine receptors present on the terminal cisternae of the sarcoplasmic reticulum. This mediates calcium release and induces contraction of the cardiomyocyte. In many common cardiovascular diseases, the t-tubule structure is compromised and can include elongation, distortion, and atrophy of the t-tubule. It remains unclear how the relationship between with the BM is affected and if BM defects can lead to uncoupling of the cardiac dyad ultimately leading to contractile dysfunction.

Cardiac arrhythmias have been reported in several cases where patients have mutations affecting BM components including *COL4A1*, *COL6A1*, and *LAMA2* (Table 2) [99,119,188–191]. These findings indicate a potential link between BM disruption and the receptors that are involved in calcium transport and subsequent myofilament contraction. These receptors are structurally coupled to each other in an organisation called the cardiac dyad (Figure 4). This dyad forms between t-tubules, an invagination of the sarcolemma that allows voltage gated calcium channels to sit in close proximity to ryanodine receptors on the sarcoplasmic reticulum (Figure 4A) [192]. The cardiac dyad is necessary for propagating action potentials and the contraction and relaxation of cardiomyocytes via calcium-induced calcium release [193]. Unsurprisingly, disruption of t-tubule architecture is a pathological feature of heart failure and is associated with impaired contractile function (Figure 4B) [194]. Interestingly, immunostaining of human myocardium revealed that collagen IV is localised to t-tubule openings [195]. While this may suggest a role for the BM and collagen IV in maintenance of the dyad and t-tubule structure, there has been no direct investigation of this. To the best of our knowledge, the formation of the cardiac dyad and the maintenance of t-tubule architecture has not yet been explored in a model of direct BM disruption.

The disorganisation of the cardiac dyad, in particular due to structural changes to the t-tubule, impairs regulation of Ca^2+^ movement throughout the cardiomyocyte and has been implicated in the progression heart failure [196]. Defects in BM structure and composition could potentially affect the cardiac dyad structure which in turn mediates β1 adrenoceptor signalling, inducing Ca^2+^ release [197]. Impaired calcium signalling due to interstitial ECM disruption occurs post myocardial infarction, hypertrophic cardiomyopathy and heart failure as excitation–contraction coupling is lost [198,199]. Interestingly, recent work using siRNA mediated knockdown of *Col4a2* showed that the peptide canstatin, which is released by proteolytic cleavage of α2(IV), inhibits apoptosis of cardiomyocytes induced by β1 adrenoceptor activation possibly by reducing excessive intracellular Ca^2+^ release by L-type Ca^2+^ channel [200]. Reduced *COL4A2* levels also altered cardiac electrical activity and action potential [200]. While this indicates a link between collagen IV, the BM and intracellular Ca^2+^ signalling, the mechanisms linking the L-type Ca^2+^ channel and canstatin remain unclear [200]. Similarly, it also remains to be investigated if this is limited to canstatin as opposed to uncleaved collagen IV.

Within the adult heart, the BM appears to play several important roles from providing mechanical support to cardiomyocytes to assisting in excitation–contraction coupling. This has mainly been inferred from defects in BM structure in cardiac pathologies in which contractility and electrical conduction become impaired. However, whether this reflects a functional role of the BM in these aspects, or the pathological remodelling of the BM could be consequential and not a causal factor in these cardiac defects remains unknown. One main factor which has prevented the mechanistic investigation of this link has been the lack of appropriate models of BM disruption, mostly due to the embryonic lethality associated with BM component knock-out mouse models. Conditional mouse models as well as animal models that recapitulate mutations seen in patients (Table 2) will be highly informative to identify the role of the BM within the adult heart. This also extends to the use of zebrafish models which represent a powerful but so far under-used model species to investigate the role of BM proteins in heart development and function.

## Conclusions

There is now a large body of evidence highlighting how the BM and mutations in its individual components can cause a spectrum of congenital heart defects in patients and animal models. Despite this and the growing evidence of BM disruption and remodelling in various common cardiovascular pathologies, there are major unanswered questions regarding the influence of BM defects on heart development, adult cardiac function and, importantly, the underlying pathomolecular mechanisms of cardiac defects. Several studies discussed in this review have highlighted correlations between the expression and deposition of BM components, and cardiac development and cardiomyocyte differentiation. However, the difficulties associated with studying systemic knock-out models has made investigating the causal relationship problematic. Refinement of animal models such as conditional mouse models and zebrafish will stand to unlock the door towards understanding the role of the BM in cardiac biology and disease. For example, employing cardiac specific mouse knockout models of BM components would greatly accelerate the investigation of the functional role of the BM, while overcoming the difficulties associated with BM disruption and embryo viability. Another factor which has slowed progress in answering these questions is the lack of clinical association of cardiac defects with disorders relating to BM components. Patients with mutations affecting BM components often receive limited evaluation of cardiac function and/or the outcomes have not been reported. In patients where a cardiac defect is identified, almost no mechanistic analysis is performed due to the obvious impracticality of obtaining cardiac tissue to carry out this analysis. This again emphasises the need for well-designed *in vivo* models that appropriately recapitulate the human disease to shed light on the causal mechanisms. The continued and more intensive investigation of the influence of the BM and ECM on cardiac development and function will be critical to gain a sufficient in depth understanding of the fundamental biology to underpin the identification, design, and development of novel therapeutic targets for several cardiovascular diseases.

## Abbreviations

BM: basement membrane
CPC: cardiac progenitor cell
DDR1/2: discoidin domain receptor 1/2
EC: endothelial cell
ECM: extracellular matrix
EF: ejection fraction
ER: endoplasmic reticulum
FS: fractional shortening
MMP: matrix metalloproteinase
SVT: supraventricular tachycardia
TGA: transposition of the great arteries
UCMD: Ullrich congenital muscular dystrophy
